# Supplementary material for: Bacteriophages as Potential Tools for Use in Antimicrobial Therapy and Vaccine Development
Source: Pharmaceuticals (Basel). 2021 Apr 5;14(4):331. doi: 10.3390/ph14040331 (PMC8066226; doi:10.3390/ph14040331)
Supplement: Supplementary file 1 [file pharmaceuticals-14-00331-s001.pdf]

Supporting information

Table S1. Statistical classification of diseases covering the entire range of morbid conditions under 17 categories. [1].

International classification of diseases (ICD)	
**Disease category**	**ICD code**
Certain infectious and parasitic diseases	A00-B99
Neoplasms	C00-D48
Diseases of the blood and blood-forming organs and certain disorders involving the immune mechanism	D50–D89
Endocrine, nutritional and metabolic diseases	E00–E90
Mental and behavioral disorders	F00–F99
Diseases of the nervous system	G00–G99
Diseases of the eye and adnexa	H00–H59
Diseases of the ear and mastoid process	H60–H95
Diseases of the circulatory system	I00–I99
Diseases of the respiratory system	J00–J99
Diseases of the digestive system	K00–K93
Diseases of the skin and subcutaneous tissue	L00–L99
Diseases of the musculoskeletal system and connective tissue	M00–M99
Diseases of the genitourinary system	N00–N99
Pregnancy, childbirth and puerperium disorders (maternal or obstetric causes)	O00–O99
Different disorders of fetus and newborn originating in the perinatal period	P00–P96
Congenital malformations, deformations and chromosomal abnormalities	Q00–Q99

Table S2. WHO global priority list of antibiotic resistant bacteria requiring new drug development [3].

WHO priority list of pathogens		
**Type of priority**	**Bacterial pathogen**	**Antibiotic resistance^{AR}**
Critical	*Acinetobacter baumannii*	Carbapenem^{R}
	Pseudomonas aeruginosa	
	Enterobacteriaceae including: *Klebsiella pneumonia*, *Escherichia coli*, *Enterobacter* spp., *Serratia* spp., *Proteus* spp., and *Providencia* spp, *Morganella* spp	Carbapenem, 3rd generation cephalosporin^{R}
High	*Enterococcus faecium* *Staphylococcus aureus*	Vancomycin and its intermediate, methicillin^{R}
	Helicobacter pylori	Clarithromycin^{R}
	Campylobacter	Fluoroquinolone^{R}
	Salmonella spp.	
	Neisseria gonorrhoeae	3rd generation cephalosporin, fluoroquinolone^{R}
Medium	*Streptococcus pneumoniae*	Penicillin-non-susceptible
	Haemophilus influenzae	Ampicillin^{R}
	Shigella spp.	Fluoroquinolone^{R}
